# Evaluating the Surveillance System for Spotted Fever in Brazil Using Machine-Learning Techniques

**DOI:** 10.3389/fpubh.2017.00323

**Published:** 2017-11-30

**Authors:** Diego Montenegro Lopez, Flávio Luis de Mello, Cristina Maria Giordano Dias, Paula Almeida, Milton Araújo, Monica Avelar Magalhães, Gilberto Salles Gazeta, Reginaldo Peçanha Brasil

**Affiliations:** ^1^Laboratório de Doenças Parasitárias, Instituto Oswaldo Cruz (IOC)/Fiocruz, Rio de Janeiro, Brasil; ^2^Laboratório de Referência Nacional em Vetores das Riquetsioses, IOC/Fiocruz, Rio de Janeiro, Brasil; ^3^Laboratory of Machine Intelligence and Computation Models, Electronic and Computer Engineering Department, Federal University of Rio de Janeiro, Rio de Janeiro, Brazil; ^4^Secretaria de Estado de Saúde do Rio de Janeiro – SES, Rio de Janeiro, Brasil; ^5^Instituto de Comunicação e Informação Científica e Tecnologia em Saúde – ICICT, Rio de Janeiro, Brasil

**Keywords:** public health, epidemiology, spotted fever, machine-learning, decision trees, probabilistic neural networks

## Abstract

This work analyses the performance of the Brazilian spotted fever (SF) surveillance system in diagnosing and confirming suspected cases in the state of Rio de Janeiro (RJ), from 2007 to 2016 (July) using machine-learning techniques. Of the 890 cases reported to the Disease Notification Information System (SINAN), 11.7% were confirmed as SF, 2.9% as dengue, 1.6% as leptospirosis, and 0.7% as tick bite allergy, with the remainder being diagnosed as other categories (10.5%) or unspecified (72.7%). This study confirms the existence of obstacles in the diagnostic classification of suspected cases of SF by clinical signs and symptoms. Unlike man–capybara contact (1.7% of cases), man–tick contact (71.2%) represents an important risk indicator for SF. The analysis of decision trees highlights some clinical symptoms related to SF patient death or cure, such as: respiratory distress, convulsion, shock, petechiae, coma, icterus, and diarrhea. Moreover, cartographic techniques document patient transit between RJ and bordering states and within RJ itself. This work recommends some changes to SINAN that would provide a greater understanding of the dynamics of SF and serve as a model for other endemic areas in Brazil.

## Introduction

Rickettsial diseases are zoonoses caused by bacteria of the genus *Rickettsia* that are transmitted mainly by ticks to mammalian hosts and accidentally to humans. The infections produce an acute fever and systemic complications that can lead to patient death if proper treatment is not provided in time (1–3).

In Brazil, the main rickettsiosis is spotted fever (SF), and infections caused by *Rickettsia rickettsii* are considered the most serious. Moreover, other pathogenic *Rickettsia* (*R. parkeri* and *Rickettsia* Atlantic Forest strain) are also reported in the country, although these cases may or may not be confirmed (4, 5).

Spotted fever is a systemic disease with nonspecific signs and symptoms during its early stages. Throughout its course, it can be easily confused with other diseases, but a few patients develop rashes, which is the best clinical indicator (1, 3, 5–7). High lethality seems to be associated with inaccurate clinical suspicion, which affects diagnosis and treatment opportunity (3, 5, 6).

Given this scenario, it is essential to analyze the efficacy of the Sistema de Informação de Agravos de Notificação—SINAN (Disease Notification Information System) in capturing, managing, and confirming suspected human cases of SF, and for providing information for analysis of its morbidity profile, thus contributing to decision-making at the municipal, state, and federal levels in Brazil.

Evaluation of a surveillance system (SS), such as SINAN, should promote the best use of public health resources by ensuring that only important problems are under surveillance, and that the SS operates efficiently. Insofar as possible, the evaluation of a SS should include recommendations for improving quality and efficiency (e.g., eliminating unnecessary duplication; assessing information potential of the included variables). Above all, an evaluation should assess whether a system is serving a useful public health function and meeting its objectives (8).

Therefore, apart from the monitoring system evaluation model proposed by Klaucke et al. (8), it is important to use other tools to identify the strengths and weaknesses of SINAN so that preventive measures can be implemented and improvements can be made in its organization in order to capture, manage, diagnose, and treat in a timely manner suspected cases of SF, and facilitate a reversal in mortality rates of the disease.

The techniques of machine-learning (ML); promise to be useful tools for evaluating the accuracy of the SS for SF since they are better suited to dealing with a large number of variables and performing massive data analyses than a human agent. From this perspective, this paper employs ML techniques, such as data mining and probabilistic neural network analysis combined with geographical information, in order to better understand the SS of SF (SINAN) in the state of Rio de Janeiro.

## Materials and Methods

### Study Area

**Figure 1:**
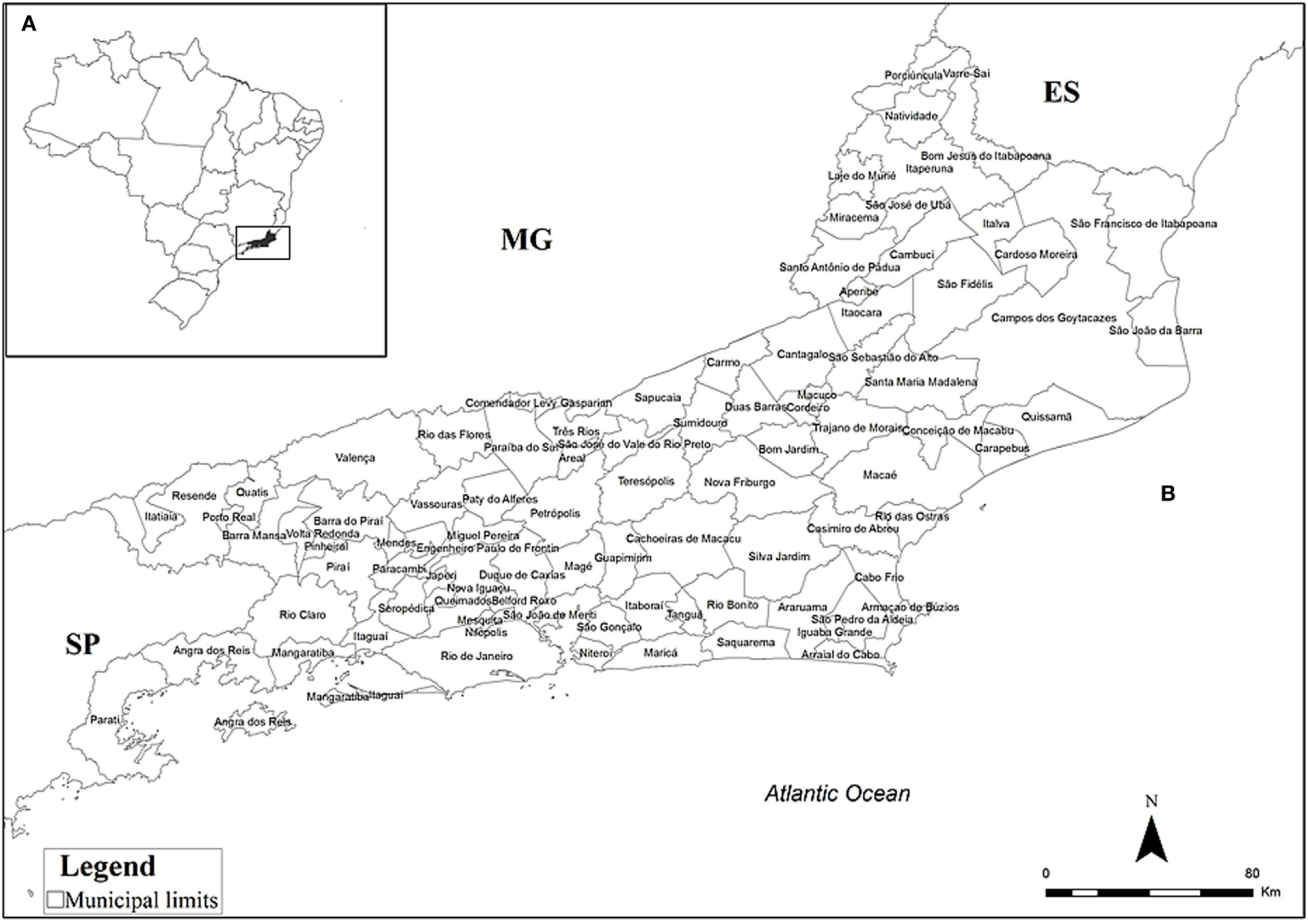
Location of the state of Rio de Janeiro, Brazil, **(A)** and its municipalities **(B)**. ES, Espírito Santo; MG, Minas Gerais; SP, São Paulo.

The state of Rio de Janeiro is located in the eastern portion of Brazil’s Southeast Region and occupies an area of 43,777.954 km^2^ divided into 92 municipalities (Figure 1). It is the fourth smallest state (by area) in Brazil, yet has the highest population density (365.23 inhabitants/km^2^) with an estimated population of 16,636,000 inhabitants and is the most urbanized state in the country, with 97% of the population living in cities (9).

### Epidemiological Data

The data presented here was obtained from SINAN and provided by the Secretaria de Estado de Saúde do Rio de Janeiro—SES/RJ (State Secretary Health of Rio de Janeiro), and encompassed notifications of suspected cases of SF between 2007 and July 2016. These data were made available with the protection of the identity of the patients; therefore, information such as names or addresses cannot be displayed at any time to comply with national ethical regulations (10).

Although cases reported to SINAN were initially separated into those confirmed by laboratory tests (PCR or Serology) and/or clinical and epidemiological nexus, unconfirmed cases and ignored cases, as reported in the corresponding epidemiological forms, all were included in the present study.

### Methods

#### Artificial Neural Networks

Classification based on probabilistic neural networks (PNN) (11), which is a feed forward neural network, was the first ML technique implemented for identifying patterns concerning the classification of reported cases into different groups of pathologies. It is a nonparametric method for classifying observations in *n* groups based on *p* qualitative and/or quantitative input variables (12–14). It implements a statistical algorithm called Kernel discriminant analysis, whereby, processes are organized to feed forward a multiple network with four types of layers: input layer, pattern layer, addition layer, and output layer (15). Through a ML process, the PNN develops the mathematical ability to perform variable predictions and correctly classify observations within pre-established categories (12–14).

In addition to its advantages over other statistical tests (11, 15), PNN was selected for implementation because of the simple and fast way by which it can process large amounts of information (11, 14, 15), the friendly way the network can be trained and its robustness to noise (14). The PNN has 31 input (*p*) and 10 output (*g*) variables. The sample space contains 528 of the 870 cases notified; the others were excluded because they did not contain information of provenance and/or lacked information regarding clinical signs. One hundred and two cases of patient records were selected for training, which contained information on area of residence (urban, peri-urban, and rural) and that confirmed 1 of the following 10 pathological categories (output) for composing the training set, as defined by SINAN: cellulitis, dengue, encephalitis, hepatitis A, leptospirosis, meningitis, other disease, SF, tick bite allergy, and virosis. The remaining 426 cases were used for testing the neural network. In this scenario, the input layer is composed of 22 clinical variables (fever, headache, abdominal pain, myalgia, nausea, rash, diarrhea, jaundice, hyperemia, hepatomegaly, petechiae, bleeding, lymphadenopathy, convulsion, necrosis, prostration, shock, coma, hemorrhage, respiratory distress, oliguria, other symptoms), 1 temporal variable (monthly reporting), 7 environmental variables [area of residence, contact with tick, capybara, dog/cat, cattle, horses, nature (forests, rivers, and waterfalls)]; and the variable hospitalization.

All variables except for the month of notification and area of residence were transformed into variables of ternary response (1 = yes or presence, −1 = no or absence, and 0 = no information) to provide values with scales easily comparable to each other. The PNN analyses were done by using the statistical package StatgraphicCenturium XVII (16).

#### Knowledge Discovery

In this work, we used another ML technique combined with data mining. Briefly, the goal was to automatically build a knowledge representation (17) by using algorithms that process combinatorial searches and discover correlations in large volumes of data. The algorithms used are associated with a technique called decision trees (18), such as: Best First Decision Tree, Decision Stump, Functional Tree, J48, Logistic Model Trees, Reduced-Error Pruning Tree, and Simple Classification and Regression (19, 20). The appropriate algorithm to be used depends on the problem being studied and its constraints, so the algorithm chosen is usually based on literature reports. However, there are no articles describing ML algorithms applied to the problem addressed by the present work. For this reason, an exhaustive test of all listed algorithms was executed. Cross-referencing of 23 clinical and seven epidemiological variables was performed in order to evaluate if a patient case might prove fatal. Cases in which the evolution was recorded as “ignored” do not contribute positively to the ML process because they introduce a component of uncertainty about the evolution of the case, and so, these cases were excluded from the sample space.

Decision trees were built and optimized using cross-validation over a *k* number of folds. In such *k*-fold cross-validation, the original sample is randomly partitioned into *k* subsamples. Among all *k* subsamples, a single one is retained as the validation data for testing the model, and the remaining *k* − 1 subsamples are used as training data. The cross validation process is then repeated *k* times (the folds), with each of the *k* subsamples used exactly once as the validation data. Then, the *k* results from the folds are averaged to produce a single estimation. This procedure was accomplished by using the free software Weka (Waikato Environment for Knowledge Analysis) (19).

#### Mapping Process

The mapping process was performed using the most relevant attributes of the previously discussed analyses and the confirmed cases of SF. The observations of the confirmed cases were studied by measures of central tendency and distribution according to case evolution: recovered, death, and ignored. At this stage, the cases recorded as confirmed by laboratories were compared with the criteria set out in the epidemiological surveillance guides for the years 2007–2016 (4, 5, 21, 22).

#### Cartographic Techniques

Finally, using the data of confirmed SF cases (*n* = 104), a study of patients spatial behavior was undertaken according to residence, infection, and medical care, using the program Terraview (23). Subsequently, this study was exported to the program ArcGis program (24), which was used to develop thematic maps for the identification of spatial patterns.

## Results

**Figure 2:**
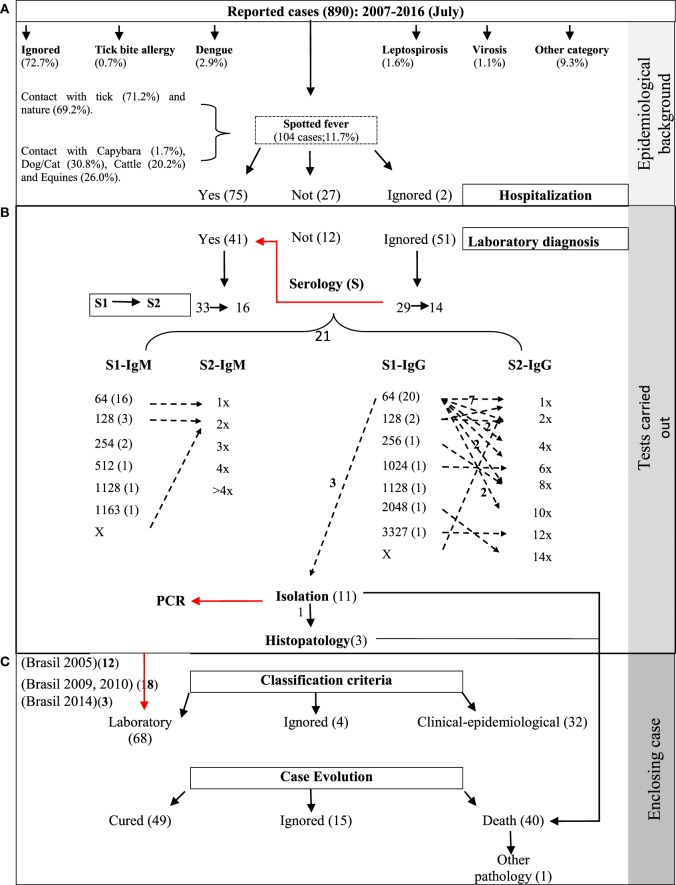
Process map for epidemiological surveillance of spotted fever (SF), 2007–2016. **(A)** Descriptive epidemiological analysis of the cases reported to SINAN and hospitalization of cases confirmed as SF. Data inconsistency (→). For example, of 51 cases without laboratory tests recorded (ignored), evidence was found in 29 using indirect immunofluorescence assay in the first sample and 14 for the paired sample. **(B)** Follow-up to laboratory techniques and serological titers confirming human cases with SF. Evidence was found for 33 cases through laboratory confirmation following the parameters established for the country (4, 5, 21, 22). Seroconversion serologic titers (→), for example, of 20 patients with IgG titers for 1:64 in the first sample (S1), seven exhibited no increase in titers (1×), two increased by a factor of four (4×), two by a factor of eight (8×), and two by a factor of 10 (10×). The zeta no number refers to one seroconversion patient. Serologic titers: 1× = 1:64, 2× = 1:128, 4× = 1:256, 6× = 1:512, 8× = 1:1,024, 10× = 1:2,048, 12× = 1:4,096, 14× = 1:8,192. **(C)** Comparative evaluation of the serological classification criteria with current technical standards (according to period) of Brazil and final clinical evolution of the patients with SF.

Among the 890 SF cases reported in SINAN in RJ, 11.7% (104) were confirmed as SF; 0.7% (6) associated with tick bite allergy; 2.9% (26) as dengue; 1.6% (14) as leptospirosis, and 10.5% (93) as other categories. In addition, 72.7% (647) of reported cases did not have a pathology category provided (Figure 2).

About 50% (437) of the reported cases involved hospitalization, but information concerning such hospitalization was available for just 181 patients; that is, there were missing data such as dates of hospitalization and discharge. Among the confirmed SF cases, 75 had been hospitalized, of which, 68 had their diagnosis confirmed by laboratory techniques and 32 by clinical-epidemiologic criteria; the criterion of classification was not recorded for four of the confirmed cases. Regarding the clinical outcome of the cases, 47.1% (49) of the patients recovered, 38.5% (40) died, and 14.4% (15), there was no information report (Figure 2).

**Figure 3:**
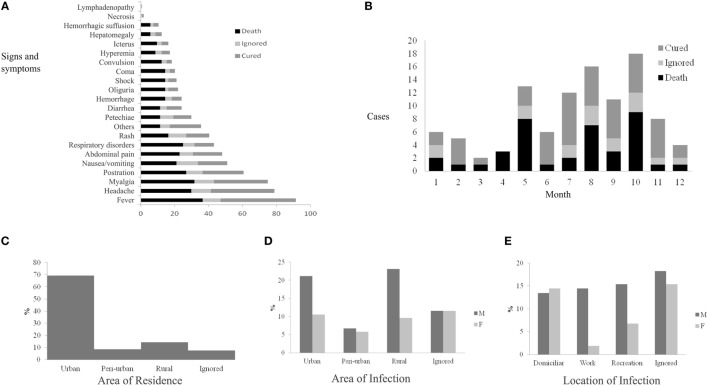
Epidemiological dynamics of spotted fever in the state of Rio de Janeiro, 2007–2016 (July): clinical signs and symptoms **(A)**, monthly distribution according to the progress of cases **(B)**, area of residence **(C)**, area of infection **(D)**, and local infection **(E)** of patients.

Among the clinical signs and symptoms, fever was present in 91.3% (95) of the confirmed cases, followed by headache, myalgia, prostration, and nausea/vomiting. The proportion of the symptoms remained relatively invariant among cases that turned into death, cases that were cured, and cases that were ignored (Figure 3).

The neural network was able to classify 38.2% (39/102) of correct instances of diagnosis (Table 1). Observe that the probabilistic bid for choosing the correct diagnosis is 10.0% since there are 10 possibilities of diseases. Although the 38.2% hit is higher than such probabilistic bid, it is still a poor classifier for determining the nature and circumstances of a diseased condition. Therefore, the PNN failed to produce good agreement in classifying cases into the pre-established disease categories using clinical and predictive environmental variables. It was observed that the Field 51 from SINAM form for recording the diagnosis was frequently not filled properly, and thus there is a lack of information. Consequently, a reduced sample was used for training the PNN (102 cases), which compromised the performance of the neural network, resulting in a low overall percentage of correct classification.

**Table 1:** Diagnosis classification using bayesian probabilistic classification neural network in the state of Rio de Janeiro.

Diagnosis	Cases	Correct instances
Cellulitis	2	0 (0.0%)
Dengue	14	4 (28.6%)
Encephalitis	1	1 (100.0%)
Hapatitis A	2	0 (0.0%)
Meningitis	3	0 (0.0%)
Leptospirosis	7	2 (28.6%)
Other disease	31	11 (35.5%)
Spotted fever	38	21 (55.3%)
Tick bite allergy	2	0 (0.0%)
Virosis	2	0 (0.0%)
Total	102	39 (38.2%)

In the analysis of clinical evolution of patients using data mining and ML, some of the algorithms had irrelevant results; the best results were obtained with the algorithms Best First Decision Tree, J48, and Reduced-Error Pruning Tree. All of the algorithms generated decision trees for identifying probable deaths with only epidemiological variables and no environmental variables.

Using only the 27 clinical variables resulted in Kappa coefficients with higher values and located completely inside the interval of substantial agreement, with the prioritized variables being: respiratory disorders, convulsion, shock, petechiae, coma, icterus, and diarrhea (Table 2).

**Table 2:** Classification of cases of spotted fever in the state of Rio de Janeiro as death or recovery using epidemiological variables and prioritized clinical variables.

Algorithm	Kappa	Correct instances (%)	Folds	Selected tree decision variables
Best First decision tree	0.2935	67.1	19	Contact with tick, cattles, woods-forest-river-waterfalls, other
J48	0.3648	70.5	22	Contact with woods-forest-river-waterfalls, other
Reduced-error pruning tree	0.3159	68.2	12	Contact with woods-forest-river-waterfalls, other
Best first decision tree	0.68	84.1	28	Respiratory disorders, convulsion
J48	0.62	80.9	8	Respiratory disorders, convulsion
Reduced-error pruning tree	0.62	80.7	4	Coma, convulsion, icterus, respiratory disorders, diarrhea

The machine learning algorithms produced six rules (Table 3) that allow deducing that the evolution of a patient’s case will be death.

**Table 3:** Prediction rules obtained by machine learning for death from spotted fever (SF) in the state of Rio de Janeiro.

Rule		Reliability (%)	Support (%)
R1	Respiratory disorders → death	63.2	61.5
R2	¬ Respiratory disorders ^ convulsion → death	85.7	15.4
R3	Coma → death	100.0	30.8
R4	¬ Coma ^ icterus ^ respiratory disorders → death	100.0	10.3
R5	¬ Coma ^ ¬ icterus ^ convulsion → death	75.0	7.7
R6	¬ Coma ^ ¬ icterus ^ ¬ convulsion ^ ¬ diarrhea ^ respiratory disorders → death	60.0	7.7

*Note that the possible consequences for patient disease are death or recovery, so the random probability of death is 50%. This means that any rule with confidence value higher than 50% is better than random choice. For each of these rules, we calculated the values of two metrics: support, which indicates the percentage of SF notification records in the sample space that endorse the rule; reliability, which indicates the percentage of SF notification records whose patients in fact died when presenting the clinical symptoms described in the rule*.

**Figure 4:**
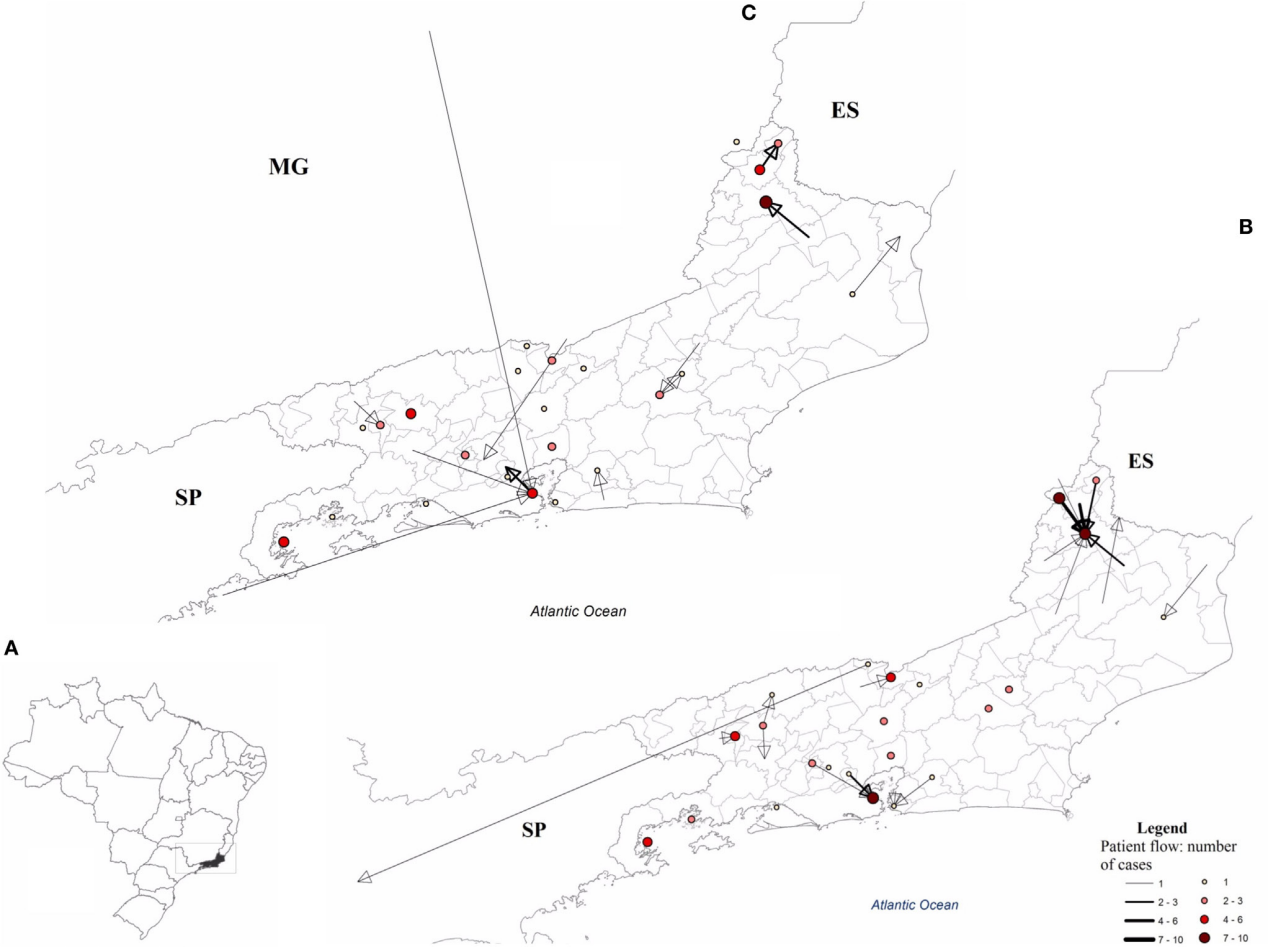
Flow of patients diagnosed with spotted fever (SF) in the state of Rio de Janeiro, 2007–2016 (July). **(A)** Area with SF patient flow, **(B)** flow between the municipality of residence and the municipality of notification, and **(C)** flow of patients from the municipality of infection to the municipality of their residence. ES, Espírito Santos; MG, Minas Gerais; SP, São Paulo.

Of the 104 cases confirmed as SF, 103 were from 25 municipalities of RJ and one from the municipality of Guarulhos, São Paulo-SP. Ninety eight of these confirmed cases were found to be for patients who reside in 15 municipalities of RJ and 1 municipality (Tombos) of Minas Gerais (MG) (Figure 4).

## Discussion

This study was not able to make a diagnostic classification of suspected cases of SF through clinical signs and symptoms using techniques of neural networks. However, ML for knowledge representation provided good results. Rash and the presence of petechiae seem to be strong indicators of SF (5–7) and were present in 40.4% (42/104) and 29.8% (31/104) of the cases, respectively (Figure 3).

Although 71.0% (74/104) of the confirmed SF patients had contact with a tick and 69.2% (72/104) had performed some activity in nature, these were not factors unique to the disease. In fact, laboratory tests confirmed cases for dengue and leptospirosis, 53.3% (8/15) and 62.5% (5/8), respectively, in which subjects had also had contact with ticks. However, contact with ticks as a historical factor of suspected SF remains important (3, 25, 26), while contact with capybaras, present in 1.7% (2/104) of cases, is not a relevant factor in suspected SF in the state of RJ (27), as established in the surveillance protocols for Brazil (4, 5, 21, 22).

This study found that some changes need to be made to the SF notification report form (28). The “ignored” alternative, which appears in various fields/variables such as sex, area of residence, all clinical signs, and symptoms, among others, makes it difficult or even impossible to achieve a deeper understanding of the epidemiological dynamics of SF and evaluate the sensitivity of SINAN, as was the case in this study. Thus, we recommend binary responses for such fields (1 or 2).

Moreover, the separation of dogs and cats in Field 34, regarding Epidemiology group, seems to be important (28), since dogs have been shown to be an important amplifier for *R. rickettsii*, Brazil (29, 30), and they usually act as hosts for several species of ticks in endemic areas of SF (31–33).

Furthermore, we emphasize the importance of instructing qualified SS professionals on how to correctly complete the epidemiological investigation forms from SINAN. We noticed, for example, that the field responsible for recording the diagnosis (Field 51) was frequently filled improperly, which caused a 72.7% (647/870) drop in the original sample size of cases. In fact, this lack of information compromised the performance of the neural networks, resulting in a low overall percentage of correct classification (45.6 and 37.3%; results not shown).

It is very important to mention that based on laboratory classification criteria (4, 5, 21, 22), only 48.5% (33/68) of the cases were confirmed by indirect immunofluorescence assay (IFA), isolation, and histopathology; the remaining cases did not meet criteria for laboratory classification (see in detail in Figure 2). Moliterno (34) previously made this same observation for confirmed cases in RJ from 2004 to 2008.

According to the technical staff of SES-RJ (*personal communication*), there was a critical situation at SINAN regarding this issue; that is, cases appearing confirmed by isolation mostly corresponded to results of PCR techniques, because there was no option on the epidemiological form for PCR (28), and so the isolation option was selected instead.

As expected, the decision trees analysis reinforced the hypothesis that epidemiological variables are not predisposing factors for the clinical evolution of the patient, as some clinical signs and symptoms are (Table 2). These results suggest that two experts on SF would agree with each other with a high frequency in their prediction of the clinical evolution (death or recovery) of cases using the same clinical variables: respiratory disorders, convulsion, shock, petechiae, coma, icterus, and diarrhea. Some of these symptoms have also been associated with more severe clinical evolution and higher case-fatality by SF (3, 7, 25, 26).

In trying to prioritize symptoms, ML algorithms produced six rules (Table 3) that allow deducing that the evolution of a particular case will be death. Recall that any rule with a confidence value higher than 50% is better than a random choice, and thus increases the probability of predicting death. Rule R4, for example, is associated with 10.3% of the sample space with 100.0% confidence; in other words, the patient will die if he has coma or convulsion and also if he has respiratory disorders with or without icterus. This analysis produced intermediate Kappa coefficient values, located at the border between the classes seen as in moderate agreement and substantial agreement (35).

There is a dynamic flow of patients among RJ municipalities and bordering states (Espírito Santo, Minas Gerais, and São Paulo), which requires future work to integrate a more detailed spatial component of the sites of infection for a greater understanding of the epidemiological dynamics of SF.

Overall, the findings here are of the utmost importance to SINAN and the SS for SF. They indicate that changes to the epidemiological form for SF are needed, that qualification of SS personnel should be improved, and that pilot studies should be established on sensitivity, focused in areas with a greater number of cases as well as epidemiological silent areas of the state of RJ.

Given the low quality of the SF case data in SINAN for the state of RJ, the artificial neural networks were not able to generate robust predictive projections. Therefore, we recommend the selection of a set of municipalities with greater epidemiological burdens of SF in RJ for future prospective study applying these techniques.

Since some diagnostic categories are very rare, for example, encephalitis, and occur only a few times in the data set, it would be advisable to limit the exit space of the PNN to more frequent and related groups of pathologies, or to do so alone with SF and other pathologies. Comparative studies with other statistical tests are needed, such as with Linear and Quadratic Discriminant Analysis.

## Author Contributions

DL—contributed to the concept and design; DL and FM—contributed to the design and application of M-L techniques and DL and CD with cartographic techniques; CD, PA, and MA with acquisition of the epidemiological information; FM, GG, and RB—contributed to concept and design of the research project, data acquisition, and interpretation of results. All authors contributed to critically revising the manuscript for important intellectual content and final approval of the version to be published. All authors are in agreement to be accountable for all aspects of the work and in ensuring that questions related to the accuracy or integrity of any part of the work have been appropriately investigated and resolved.

## Conflict of Interest Statement

The authors declare that they have prepared the manuscript in accordance with the standards of the journal, have the exclusive responsibility for the accuracy and correctness of the contents of the article submitted, and declare that they have no conflicts of interest.
